# Impact of Meniscus Repair in Conjunction With Anterior Cruciate Ligament Reconstruction on Functional Outcomes at Six Months

**DOI:** 10.7759/cureus.54999

**Published:** 2024-02-26

**Authors:** Sanjay Soni, Saptak P Mankad, Dhruv Sharma, Krunal Patel, Hemant Soni, Manan R Shroff, Shivam Sharma, Preya Rana, Tanishq S Sharma, Hardil P Majmudar

**Affiliations:** 1 Orthopedic Surgery, Shree Krishna Hospital and Medical Research Centre, Bhaikaka University, Anand, IND; 2 Internal Medicine, Dev Medical Hospital, Vadodara, IND; 3 Orthopaedic Surgery, Shree Krishna Hospital and Medical Research Centre, Bhaikaka University, Anand, IND; 4 Orthopaedic Surgery, Medical College Baroda, Vadodara, IND; 5 Pediatric Cardiac Surgery, Bhanubhai and Madhuben Patel Cardiac Centre, Shree Krishna Hospital and Medical Research Centre, Anand, IND; 6 Community Medicine, SAL Institute of Medical Sciences, Ahmedabad, IND; 7 General Surgery, ND Desai Medical College and Hospital, Nadiad, IND

**Keywords:** meniscal repair, meniscal tear, anterior cruciate ligament injury, lysholm knee score, anterior cruciate ligament reconstruction

## Abstract

Background and aim

Anterior cruciate ligament (ACL) injuries often occur along with menisci tears. ACL reconstruction with meniscectomy has long been the preferred technique for such injuries; however, it has been postulated to increase the chances of osteoarthritis (OA). Therefore, recent techniques have involved preserving menisci while reconstructing ACL to prevent OA and improve overall functional outcomes.

This study aimed to evaluate the functional outcomes of arthroscopic meniscal repair performed concurrently with ACL reconstruction at six months post-surgery.

Methodology

We conducted a cross-sectional study at a tertiary care center after getting appropriate ethics committee approval. A total of 67 participants who met the inclusion and exclusion criteria were enrolled in the study after obtaining informed consent. Their demographics were recorded retrospectively from hospital records, while their Lysholm Knee Score (LKS) responses were collected prospectively during their sixth-month follow-up visit to our department. Analysis was done using Microsoft Excel. Appropriate statistical tests including chi-square, analysis of variance (ANOVA), and independent t-tests were applied to keep an alpha of 0.05.

Results

We found that the mean age of participants was 35 years. The mean LKS of patients who underwent isolated ACL reconstruction (ACLR) was 86.02 ± 9.38. For those who underwent ACLR plus meniscus repair (MR), the mean LKS was marginally higher at 87.4 ± 7.41 during their sixth-month follow-up, with a *P*-value of 0.27. Furthermore, the mean LKS of patients who underwent ACLR plus meniscectomy was 86 ± 10.48. Comparing the means of all three groups revealed no statistical difference among any surgical approach with a *P*-value of 0.69. A total of 33 (49.25%) participants achieved an LKS falling within the *Good* category (84-94). Comparing between three surgical groups and their LKS categories also revealed no statistical difference with a *P*-value of 0.7.

Conclusions

Short-term functional outcomes in patients undergoing ACLR or ACLR plus MR using patient-reported knee scores like LKS demonstrate favorable outcomes but fail to demonstrate statistical significance. On a longer follow-up period, a reduction in the prevalence of OA is a possibility with the preservation of menisci; however, conflicting evidence in the literature about the approach to ACL injuries with menisci involvement warrants large-scale randomized controlled trials to decide upon the standard of care.

## Introduction

It is commonly observed that menisci tears co-exist with the anterior cruciate ligament (ACL) in acute injuries to the knee [1]. However, diagnosing menisci injuries on magnetic resonance imaging (MRI) is difficult, and therefore, these conjugal menisci injuries reveal themselves during surgeries for ACL reconstruction (ACLR). In patients diagnosed with ACL injury, longitudinal studies have demonstrated that they are at increased risk of osteoarthritis (OA) [2,3]. When ACL injuries are present alone, the prevalence of long-term OA is found to be at 31%; however, when concomitant menisci lesions are present, the risk increases, and the prevalence of OA is found to be at 59% [4].

It has also been well established that meniscectomy during ACLR increases the chances of OA in patients; therefore, menisci preservation through repair has garnered increased interest among surgeons [5]. There are various techniques to repair menisci that have been studied and described like open and arthroscopic procedures, outside-in sutures, inside-out sutures, and all-inside sutures. There have been studies that demonstrate good short- and medium-term results after these suturing procedures with various factors influencing the outcome of menisci repair (MR) and ACLR being one of them [6]. The success rate of MR was reported between 75% and 92% [5]. Due to the multitude of factors involved, there have been higher reoperation rates in patients who undergo meniscal repair [4].

Functional outcomes for ACL and meniscal injuries can be assessed using the International Knee Documentation Committee (IKDC) score, Lysholm Knee Score (LKS), and Tegner Activity Scale (TAS). LKS imposes minimal respondent and administrative burden, and it has been recognized as a reliable tool for assessing ligament and meniscal injuries [7]. There is little evidence when it comes to choosing between ACLR and ACLR plus MR based on short- and long-term functional outcomes. To the best of our knowledge, there is scant evidence in the Indian population to study the effect of MR in association with ACLR, particularly short- and long-term impacts.

In this cross-sectional study, we investigate the debated management options for ACL tears, specifically focusing on the functional outcomes using the LKS for ACLR versus ACLR with MR versus ACLR with meniscectomy.

## Materials and methods

We carried out a cross-sectional study at a busy tertiary care center in western India. After appropriate ethics committee approval, we recruited participants who had undergone either isolated ACLR, ACLR with MR, or ACLR with meniscectomy between 2023 and 2024 and were presented to the orthopedics department for their sixth-month follow-up. After collecting participants’ written informed consent, those who satisfied the inclusion criteria were enrolled. Patients with a confirmed diagnosis of ACL injury or ACL with meniscal injury who underwent any of the aforementioned procedures were included in the study while those who had a history of previous knee injury or knee comorbidity gave negative consent for the study or those with inadequate data were excluded from the study. Adhering to these criteria, we collected data from 70 patients out of which three were excluded due to inadequate patient-reported data, leaving 67 patients in our dataset. Data regarding demographics, current symptoms, diagnosis, type of surgery, and LKS were collected using a patient-reported questionnaire and hospital records. LKS is a validated tool to assess functional outcomes in patients with ligaments and meniscal injury.

The procedures were performed under due tourniquet control. Spinal anesthesia was administered to all participants during the surgery with intraoperative antibiotic coverage. ACLR was primarily done using quadrupled hamstring autografts and peroneus longus autografts. Upon arthroscopy, the involvement of the menisci was evaluated visually and confirmed with a probe. MR was mainly done using all-inside, inside-out, outside-in, or combining either or all approaches as per the surgeon’s judgment. The surgeon and their team followed appropriate protocols during the surgery. Specific emphasis was given while harvesting the graft and putting it into an antibiotic solution for preservation as well as while creating the femoral tunnel and ensuring its patency and position through guidewire. Additionally, care was taken while creating the tibial tunnel so that the graft passage was smooth. Finally, the graft was visually inspected for any signs of impingement after placement. Post-fixation, the negative anterior drawer test, Lachmann test, and pivot shift tests were confirmed, with intact distal vasculature.

**Figure 1:**
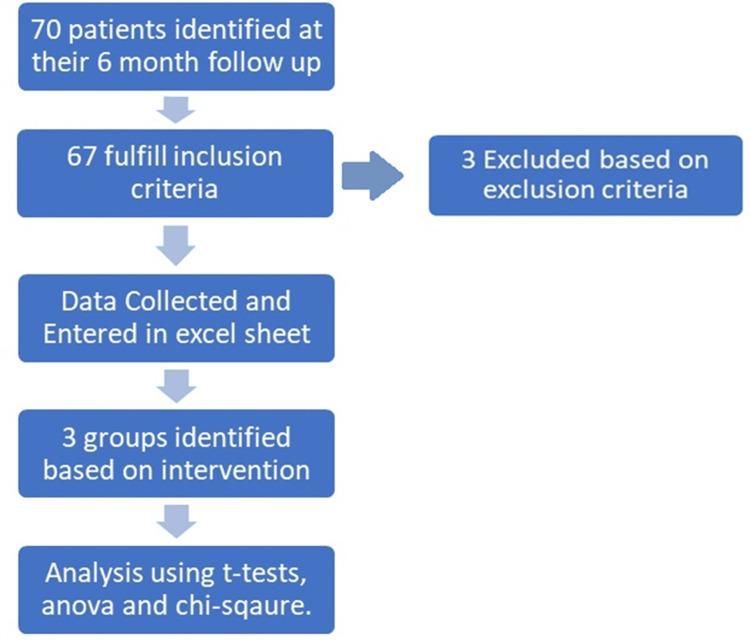
Flowchart showing study methodology. Image credit: Sanjay Soni. ANOVA, analysis of variance

The data were entered into an Excel sheet and analyzed using Excel and IBM SPSS Statistics for Windows, Version 27.0 (IBM Corp, Armonk, NY). Continuous variables were analyzed using appropriate analytical tests like independent t-test and single-factor one-way analysis of variance (ANOVA), whereas categorical variables were analyzed using chi-square tests. The results were interpreted with a significance level set at alpha 0.05, and only those below this threshold were considered statistically significant (Figure 1).

## Results

In our study of 67 participants, we categorized them primarily into three groups based on the treatment they received: patients who underwent isolated ACLR, patients who underwent ACLR plus MR, and patients who underwent ACLR plus meniscectomy. We found that the mean age of all participants was 35.13 ± 11.62 years. Our dataset predominantly consisted of 59 (88.05%) and 8 (11.94%) females (Table 1).

**Table 1:** Demographics. SD, standard deviation

Demographics		*P*-value
Age (years), mean (SD)		
Males	34.19 (11.57)	
Females	42.12 (12.28)	
All participants	35.13 (11.62)	
Gender, *n* (%)		
Males	59 (88.05)	0.46
Females	8 (11.94)	

We found that 36 (53.73%) were diagnosed with ACL injury compared to 23 (34.32%) and 8 (11.94%) who were diagnosed with ACL plus medial meniscal injury and ACL plus lateral meniscal injury, respectively. Isolated ACLR was performed in 36 (53.73%) participants, ACLR plus MR in 25 (37.31%) participants, and ACLR plus meniscectomy in 6 (8.95%) participants. Furthermore, among those diagnosed with a medial meniscal injury in addition to an ACL injury, 19 (82.6%) underwent ACLR with medial MR, while only 4 (17.4%) underwent meniscectomy. Among those diagnosed with ACL plus lateral meniscal injury, 6 (75%) underwent lateral meniscal repair, while 2 (25%) underwent lateral meniscus meniscectomy (Table 2).

**Table 2:** Characteristics of study participants. ACL, anterior cruciate ligament; ACLR, anterior cruciate ligament reconstruction; MR, meniscal repair; M, meniscectomy

Diagnosis		*n*	%
Isolated ACL injury		36	53.73
ACL plus medial meniscus injury		23	34.32
ACL plus lateral meniscus injury		8	11.94
Type of surgery			
ACLR		36	53.73
ACLR + MR	Medial MR	19	28.35
	Lateral MR	6	8.95
	Total	25	37.31
ACLR + M	Medial M	4	5.97
	Lateral M	2	2.98
	Total	6	8.95

The mean LKS in patients who underwent isolated ACLR was 86.02 with a standard deviation (SD) of 9.38. In comparison, those who underwent ACLR plus MR reported a mean LKS of 87.4 with an SD of 7.41, and those with ACLR plus meniscectomy reported a mean (SD) LKS of 86 (10.48). After applying single-factor ANOVA to compare the means of the three groups, the calculated *P*-value was 0.69, indicating no significance. Similarly, when comparing isolated ACLR versus ACLR with MR using an independent t-test, the *P*-value was also insignificant at 0.27 (Table 3).

**Table 3:** Shows Lysholm knee scores among groups. ACL, anterior cruciate ligament; ACLR, anterior cruciate ligament reconstruction; MR, meniscal repair; M, meniscectomy; SD, standard deviation; LKS, Lysholm knee score

Type of surgery	Mean (SD)			*P*-value
ACLR	86.02 (9.38)			0.69
ACLR + MR	87.4 (7.41)	Medial MR	88.21 (6.94)	
		Lateral MR	84.83 (8.95)	
ACLR + M	86 (10.48)	Medial M	82.75 (11.7)	
		Lateral M	92.5 (3.53)	
Category	LKS category across all participants (*n *= 67)			
	n	%		
Excellent (95-100)	16	23.88		
Good (84-94)	33	49.25		
Fair (65-83)	18	26.86		
Poor (<64)	0	0		
Category	LKS category among participants as per groups			0.70
	ACLR, *n* (%)	ACLR + MR, *n* (%)	ACLR + M, *n* (%)	
Excellent (95-100)	9 (25)	5 (20)	2 (33.3)	
Good (84-94)	16 (44.44)	15 (60)	2 (33.3)	
Fair (65-83)	11 (30.56)	5 (20)	2 (33.3)	
Poor (<64)	0	0	0	

**Figure 2:**
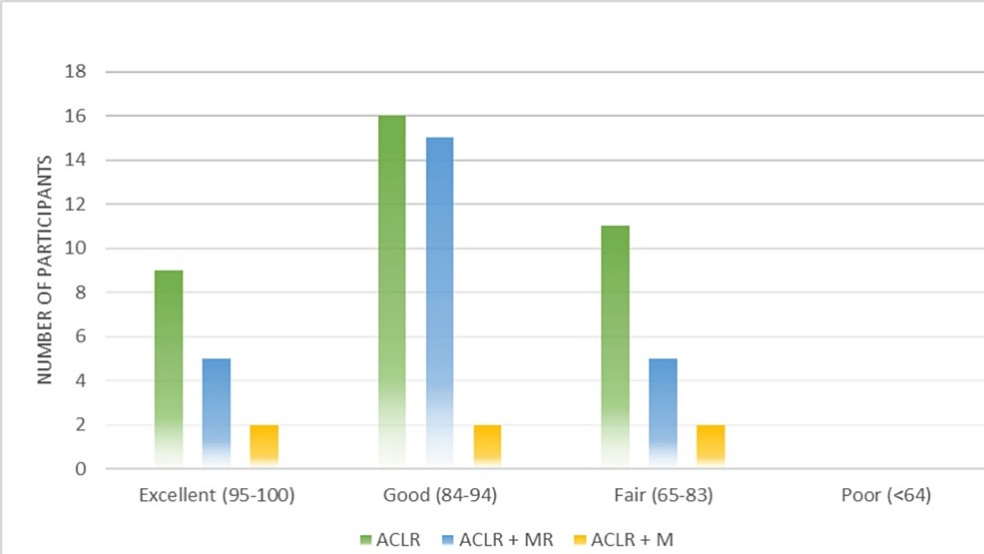
Showing graphical representation of the LKS category among groups according to their surgeries. ACL, anterior cruciate ligament; ACLR, anterior cruciate ligament reconstruction; MR, meniscal repair; M, meniscectomy

When grouping all participants according to their LKS, 16 (23.88%) reported excellent scores (95-100), the majority of the participants (33, 49.25%) reported good scores (84-94), 18 (26.86%) reported fair scores (65-83), and no one reported poor scores, i.e., <64 (Figure 2).

Furthermore, chi-square testing for categorical variables revealed a *P*-value of 0.7, indicating no significance.

## Discussion

ACL injuries are a predisposing factor for the long-term development of radiographic OA. It has been long debated and studied extensively whether reconstruction of the ACL would prevent or reduce OA in the long run due to various factors that interplay in causing OA after an injury [8-10]. However, there is plenty of evidence when it comes to ACLR with meniscectomy, proving that removal of menisci does accelerate OA [6,11]. Therefore, techniques have been developed to perform ACLR and preserve the meniscus to improve the risk of OA and functional outcomes.

In this study assessing functional outcomes using LKS in patients who underwent ACLR, ACLR plus MR, or ACLR plus meniscectomy, it was found that statistically, there was no difference in mean LKS between all three groups. A systematic review by Sarraj et al. reported a mean LKS of 86.71 ± 7.77 in 3,425 patients undergoing ACLR with MR, a finding closely aligned with the results of our study. In patients undergoing ACLR with partial meniscectomy (PM), Sarraj et al. reported a mean LKS of 80.3 ± 8.57 in 1,910 patients, a result falling into a different LKS category compared to our study. This discrepancy could be attributed to the limited dataset of six patients in our study's group [12]. Chalmers et al., in their systematic review, observed that ACLR had a reported mean LKS of 88.7, a finding closely resembling what this study reports and falling within a similar category [9].

The majority of our patients’ LKS consistently fell in the good and excellent category regardless of the surgery. This is in accordance with other studies by Sharma et al. and Pathak et al. [13,14]. Furthermore, as observed by Rodriguez et al., a significant proportion (49%) of our study population reported good results, closely aligning with their finding of 55% [15]. This could be explained by the fact that reconstruction and/or repair of the menisci stabilizes the joint and prevents further damage, translating into better patient functional outcomes. The inside approach was the most commonly used technique among our participants, aligning with what is evidenced in the literature [16].

In their review, Lieter et al. argued that meniscal surgery, especially medial MR or meniscectomy is a strong predictor of OA. They further reported that reconstructed knees had a higher chance of developing OA as compared to nonreconstructed knees [17]. This is in contrast to the general consensus of non-operated injured knees being more susceptible to OA [2,3]. Some recent studies even further suggest ACLR failure due to meniscectomy and the absence of menisci [18,19].

At a short-term follow-up of six months, our study reported no difference in functional outcomes. In their retrospective study, Casp et al. reported similar findings, with no significant differences in strength, jumping performance, or patient-reported knee scores between groups that underwent isolated ACLR, ACLR plus MR, or ACLR plus PM. They concluded that a return to sports is an ideal indicator of a successful outcome and should be assessed over longer follow-up periods, as six months is considered a very short duration [20]. We agree with this statement and believe that longer follow-up periods are necessary to evaluate the quality of life and knee scores in such patients.

Limitations

The authors are aware of the limitations of this study. This study could benefit from a larger sample, thus enabling the interpretation of results with greater confidence. Other functional knee scores could be incorporated in subsequent follow-up periods to enhance the reliability of outcome assessment. Additionally, the absence of control cohorts and preoperative LKSs makes it challenging to evaluate improvements in outcomes.

## Conclusions

The concept of preserving the meniscus originates from the belief that menisci act as a cushion between joint surfaces, reducing their contact and thereby preventing joint degeneration. Short-term outcomes in terms of quality of life using patient-reported knee scores fail to demonstrate superior outcomes between ACLR and ACLR plus MR. On a longer follow-up period, a reduction in the prevalence of OA could be seen. However, conflicting evidence in the literature regarding the approach to ACL injuries with meniscal involvement highlights the need for large-scale randomized controlled trials to establish the standard of care.
